# Single-cell analysis reveals the spatiotemporal effects of long-term electromagnetic field exposure on the liver

**DOI:** 10.3389/fcell.2025.1579121

**Published:** 2025-06-27

**Authors:** Mingming Zhang, Zhichun Lv, Lingping Zhao, Quan Zeng, Yunqiang Wu, Junnian Zhou, Jiafei Xi, Xuetao Pei, Haiyang Wang, Changyan Li, Wen Yue

**Affiliations:** Beijing Institute of Radiation Medicine, Beijing, China

**Keywords:** electromagnetic field, liver function, single-cell transcriptome, hepatic zonation, single-cell analysis

## Abstract

**Introduction:**

Artificial electromagnetic fields (EMFs) can impair the functions of several organs. The impact of long-term artificial EMF on the liver, the synthetic and metabolic center of the body, has become concerning. The aim of this study was to systematically evaluate the effect of long-term EMF exposure on the liver.

**Methods:**

Mice were exposed to 2.45 GHz EMF daily for up to 5 months, and serum liver function test, lipidomic analysis, and histological analysis were performed to detect the general impact of EMF on the liver. Furthermore, EMF-induced liver transcriptome variations were investigated using single-cell RNA sequencing and a spatiotemporally resolved analysis.

**Results:**

Different hepatic cells exhibited diverse sensitivities and response patterns. Notably, hepatocytes, endothelial cells, and monocytes showed higher sensitivity to electromagnetic radiation, with their lipid metabolic functions, immune regulation functions, and intrinsic functions disturbed, respectively. Moreover, transcriptomic alterations were predominantly observed in the hepatocytes and endothelial cells in peri-portal regions, suggesting a zonation-related sensitivity to EMF within the liver.

**Conclusion:**

Our study provided a spatiotemporal visualization of EMF-induced alterations in hepatic cells, which ultimately elucidated the biological effects of EMF exposure.

## 1 Background

Electromagnetic fields (EMFs) are omnipresent on earth. However, with the extensive application of wireless communications, military or commercial radars, and microwave ovens, the public has been increasingly and persistently exposed to artificial EMFs (Wiart et al., 2019). Long-term exposure to approximately 2.4 or 5 GHz EMF, the most common artificial EMF frequencies, can impair the functions of the nervous, productive, and hematological systems at the nonthermal level (Singh et al., 2023; Liu et al., 2021; Pacchierotti et al., 2021; Jaffar et al., 2019; Busljeta et al., 2004), resulting in sleep disorders (Liu et al., 2021; Huber et al., 2002; Danker-Hopfe et al., 2020), spatial learning and memory decline (Hao et al., 2023), decreased sperm motility and viability (Jaffar et al., 2019; Liu et al., 2015; Houston et al., 2019), and even disturbances in red blood cell maturation (Busljeta et al., 2004). Therefore, concerns over the safety of electromagnetic radiation are growing and scientific research studies are being conducted to explain them.

The potential impact of long-term exposure to artificial EMF on the liver, the synthetic and metabolic center of the body, has also emerged as a significant concern. Multiple studies have reported that electromagnetic radiation can induce oxidative stress in liver tissues, leading to double-stranded DNA breaks and morphological and biochemical changes (Okatan et al., 2019; Çelik et al., 2016; Koyu et al., 2009; Alkis et al., 2021). However, whether long-term EMF affects liver functions is unclear. In particular, the liver is a complex organ characterized by diverse cellular composition and zonated gene expression. Therefore, whether different hepatic cells respond differently to EMF and whether EMF sensitivity varies among different regions within the liver are worthy of in-depth research.

This study was designed to systematically evaluate the biological effects of long-term EMF exposure on the liver, particularly the underlying cellular and molecular alterations. To achieve this, we established a long-term radiation animal model by exposing mice to 2.45 GHz EMF daily for 5 months. Serum liver function test, lipidomic analysis, and histological analysis of the liver were performed to assess liver function alterations. Considering that single-cell and spatial omics have unveiled the functional heterogeneities and spatial divisions of hepatic cells, which helped elucidate the mechanisms underlying liver homeostasis (MacParland et al., 2018), disease (Zhou et al., 2022; Zhang et al., 2020; Chen et al., 2023), and regeneration (Pepe-Mooney et al., 2019; Wang et al., 2024; Chembazhi et al., 2021), we conducted a thorough spatiotemporal investigation of liver tissues using single-cell RNA sequencing (scRNA-seq).

Here, we uncovered a spectrum of sensitivities and response patterns among different hepatic cells. Compared with other cells, hepatocytes, endothelial cells, and monocytes showed higher sensitivity to electromagnetic radiation, with their lipid metabolic functions, immune regulation functions, and intrinsic functions disturbed, respectively. Moreover, the transcriptomic alterations were predominantly observed in the hepatocytes and endothelial cells in peri-portal regions, suggesting a zonation-related sensitivity to EMF within the liver. The results of this study revealed the spatiotemporal features of EMF-induced alterations in hepatic cells, which expanded our understanding of the biological effects of EMF, and provided novel insights into the response patterns of liver to environmental health risk factors.

## 2 Methods

### 2.1 Animals

Adult C57BL/6J male mice (*n* = 16, 24 ± 1 g, 10–12 weeks) were purchased and housed in an isolated ventilated cage barrier facility at the experimental animal center (Beijing, China). The mice were maintained on a 12 h light/dark cycle at 22–26°C with sterile pellet food and water *ad libitum* for at least 7 days for habitation. The mice were randomly divided into four groups: (1) 90 days exposure (3m-EMF), (2) 150 days exposure (5m-EMF); (3) 90 days control (3m-Ctrl), and (4) 150 days control (5m-Ctrl). All animal protocols and facilities used in this study were approved by the Institutional Animal Care and Use Committee in compliance with the Beijing Medical Experimental Animal Care Commission (IACUC-DWZX-2024-561).

### 2.2 Microwave exposure system

The exposure system and animal placement were same to reported study (Tan et al., 2017; Wang et al., 2013). The radiation equipment was placed in an electromagnetic shield chamber (7 m × 6.5 m × 4 m) with pyramidal microwave absorbers covering the inner walls to minimize reflections. The experimental continuous wave was generated using a klystron amplifier (model JD 2000, Vacuum Electronics Research Institute, Beijing, China), which is suitable for generating waves in the S-band with a frequency of 2.45 GHz. The mouse container was fixed in the chamber and a signal amplifier was set on the ceiling (1.4 m high) to stabilize the EMF radiation and ensure that the mice were uniformly radiated. The bottom of the container was covered with an organic wave-absorbing material to avoid reflection, and it included single compartments for each mouse to prevent mice overlapping. The mice were brought into the chamber daily and exposed to 2.45 GHz EMF with an average field power density of 35 mW/cm^2^ for 6–8 min; the calculated mouse exposure SAR value was 15 W/kg. Control mice were processed in parallel without radiation (sham-exposed) to avoid divergence caused by psychophysiological effects.

### 2.3 scRNA-seq sample collection and preparation

For scRNA-seq, six mice were sacrificed, and liver tissues were collected. Single-cell suspensions were generated using a liver dissociation kit (130-105-807, Miltenyi Biotec, Bergisch Gladbach, Germany), allowing for a high yield of non-parenchymal mouse hepatic cells. Single-cell transcriptomic amplification and library preparation were conducted using the Chromium Single Cell 3′Reagent Kit v3 (10× Genomics, Pleasanton, CA, United States) in accordance with the manufacturer’s instructions. Sequencing was performed using an Illumina NovaSeq6000 platform. Approximately 64,000 cells were collected for data processing. The remaining liver tissues were fixed in 4% paraformaldehyde and embedded in paraffin.

### 2.4 Single-cell data quality control and basic processing

Raw data were processed using the 10× Genomics Cell Ranger pipeline (v6.0) to produce BCL files in accordance with the manufacturer’s instructions. The BCL files were demultiplexed using the mkfastq command into FASTQ files, which were then aligned to the *Mus musculus* genome reference (GRCm38/mm10, UCSC). Samples from the 3m-Ctrl, 3m-EMF, 5m-Ctrl1, 5m-Ctrl2, 5m-EMF1, and 5m-EMF2 groups were integrated using the RunHarmony command in the R Harmony package (v0.1.1). Cells with less than 200 genes or 500 reads were excluded. Cells with mitochondrial gene transcript percentages >20% were removed. All data were log-normalized (“NormalizeData” function) and scaled (“ScaleData” function) with recommended default parameters. Based on the top 2000 high-variable genes selected by the “FindVariableFeatures” function, the first 30 PCs were confirmed using the “RunPCA” function for clustering and dimension reduction. The interference of differential genes between the control groups was excluded to reveal biologically reproducible rather than random events.

### 2.5 RNA extraction and quantitative reverse transcription PCR (RT-qPCR)

For gene expression and RNA extraction, snap-frozen liver samples were dissolved in TRIzol reagent and homogenized with TissueLyser. RNA was further extracted according to the Qiagen RNEasy Mini kit protocol and transcribed into cDNA. The primers used in this study were as follows: *Pck1*: forward 5′-CTGCATAACGGTCTGGACTTC-3′ and reverse 3′-CAGCAACTGCCCGTACTCC-5′; *Bhmt*: forward 5′-TTAGAACGCTTAAATGCCGGAG-3′ and reverse 3′-GATGAAGCTGACGAACTGCCT-5′; *Acat2*: forward 5′-CCCGTGGTCATCGTCTCAG-3′ and reverse 3′-GGACAGGGCACCATTGAAGG-5′; *Cyp2e1*: forward 5′-CGTTGCCTTGCTTGTCTGGA-3′ and reverse 3′-AAGAAAGGAATTGGGAAAGGTCC-5′; *Akrlc6:* forward 5′-CAGACAGTGCGTCTAAGTGATG-3′ and reverse 3′-CGGATGGCTAGTCCTACTTC CT-5′*; Hmgcs2:* forward 5′-AGAGAGCGATGCAGGAAACTT-3′ and reverse 3′-AAGGATGCCCACATCT TTTGG-5′.

### 2.6 Data analysis and graphics

Analytical procedures and graphics were performed and plotted using *R* (v4.2.2) and GraphPad Prism10. After identifying cell neighbors, clustering cells, and visualizing cell clustering with the “FindNeighbours,” “FindClusters,” and “RunUMAP” functions, respectively, we applied the “FindAllMarkers” function to identify representative genes of each cluster. Subsequently, “FindMarkers” was employed to filter significant differentially expressed genes (DEGs) between different conditions within the cell populations. The parameters for filtering significant DEGs were adj.*p* value <0.05 with the Wilcox method. The DEGs between two ctrl groups were considered as interfering DEGs and were excluded from further analysis. The “AUCell” package (v1.20.2) was used to evaluate the scores of function-related pathways (the M2 mouse collection in MSigDB) in liver immune cells.

### 2.7 Histology and immunostaining

Formalin-fixed, paraffin-embedded tissue sections were subjected to histological analysis. For hematoxylin and eosin (H&E) staining, slides were deparaffinized, rehydrated, and stained in accordance with standard protocols.

For immunofluorescence (IF) staining, the 4-μm paraffin-embedded sections were deparaffinized, and antigen retrieval was performed by microwaving for 10 min. Then the sections were permeabilized with 0.2% Triton X-100 before blocking with 10% donkey serum (MB4516-1, meilunbio, Dalian, China, diluted in PBS) for 1 h in RT. Subsequently, sections were incubated overnight at 4 °C with the following primary antibodies respectively: Ki67 (9129s, Cell Signaling Technology, Danvers, MA, United States, 1/400), F4/80 (30325s, Cell Signaling Technology, 1/400), Ck19 (ab52625, Abcam, Cambridge, United Kingdom, 1/500), CD3E (78588, Cell Signaling Technology, 1/200), CD20 (ab64088, Abcam, 1/400), CD31 (AF3628, R&D systems, Minneapolis, MN, United States, 10 μg/mL), Clec4f (PA5-47396, Thermo Fisher Scientific, 5 μg/mL) and HNF4a (3113s, Cell Signaling Technology, 1/200). Finally, sections were incubated at room temperature for 30 min with fluorescence-labeled secondary antibodies (Donkey anti-Rabbit Alexa Fluor 488, A-21206; Donkey anti-Goat Alexa Fluor 488, A-11055; Donkey anti-Rabbit Alexa Fluor 568, A10042; Thermo Fisher Scientific, Waltham, MA, United States; 1/400).

For immunohistochemical staining, after deparaffinization and antigen retrieval, sections were treated with hydrogen peroxide and permeabilized with 0.25% Triton X-100. Then the sections were blocked with 10% goat serum (ZLI-9022, ZS bio, Beijing, China, diluted in PBS) for 1 h in RT and incubated with cleaved caspase 3 antibody (9,661, Cell Signaling Technology, 1/400) overnight at 4 °C. Subsequent steps were performed with 3,3′-diaminobenzidine chromogenic reaction kit (ZLI-9018, ZS bio).

Images were acquired with TissueFAXS (Tissue Gnostics GmbH, Vienna, Austria) and processed with Fiji (v2.14.0) for cell counting.

### 2.8 RNA in situ hybridization

*Saa1* was detected using an RNAscope Multiplex Fluorescent Detection Kit v2 (323100, Advanced Cell Diagnostics, Newark, CA, United States). Liver sections were deparaffinized in xylene and dehydration in ethanol. Then, slides were incubated with the RNAscope Target Retrieval Reagents (322000, Advanced Cell Diagnostics) at the boiling temperature (98°C–102°C) for 30 min, and rinsed in deionized water. Subsequently, sections were treated with the RNAscope Protease Reagent (322381, Advanced Cell Diagnostics) at 40°C for 30 min in a HybEZ hybridization system (310013, Advanced Cell Diagnostics). Hybridization with target probes (RNAscope Mm-Saa1-C2 probe, 450191-C2, Advanced Cell Diagnostics), amplifier, probe labeling, and detection (Opal 520, FP1487001KT, PerkinElmer, Waltham, MA, United States) were performed step by step according to the user manuals. IF staining for E-cadherin (AF748, R&D systems, 10 μg/mL) was performed after the hybridization.

### 2.9 Lipidomic analysis

Fatty acid profiles of liver samples (stored in liquid nitrogen) from 5m-group and 5m-control mice were analyzed using gas chromatography-triple quadrupole mass spectrometry (GC-MS/MS, GCMS-TQ8040NX, Shimadzu Corporation, Kyoto, Japan). Briefly, an appropriate amount of sample, added with methanol, 36% sodium phosphoric acid and methyl tert-butyl alcohol, was homogenized with stainless steel beads. After the mixture was shaken and centrifuged, supernatant extraction was repeated twice and combined. Subsequently, the supernatant was blown dry with nitrogen and added with BF_3_-CH_3_OH, saturated NaCl solution, and n-hexane. Subsequently, the mixture was vortexed (1 min) and centrifuged (20,000 r/min, 15 min). The upper solution was put into the injection vial for GC-MS/MS. Raw data were processed and compared between two groups according to the reported methods by Dai et al. (2015).

### 2.10 Statistical analysis

For single-cell RNA sequencing data, differential gene expression was assessed using the adjusted *P* value, which was corrected using the Bonferroni method to determine statistical significance.

### 2.11 Data availability

Sequencing reads and single-cell expression matrices have been deposited in NCBI’s Gene Expression Omnibus and are accessible through GEO series accession number GSE271028.

### 2.12 Code availability

All code generated for analysis is available from the authors upon requests.

## 3 Results

### 3.1 Functional and histological analyses of mouse liver under persistent exposure to 2.45 GHz EMF

**FIGURE 1:**
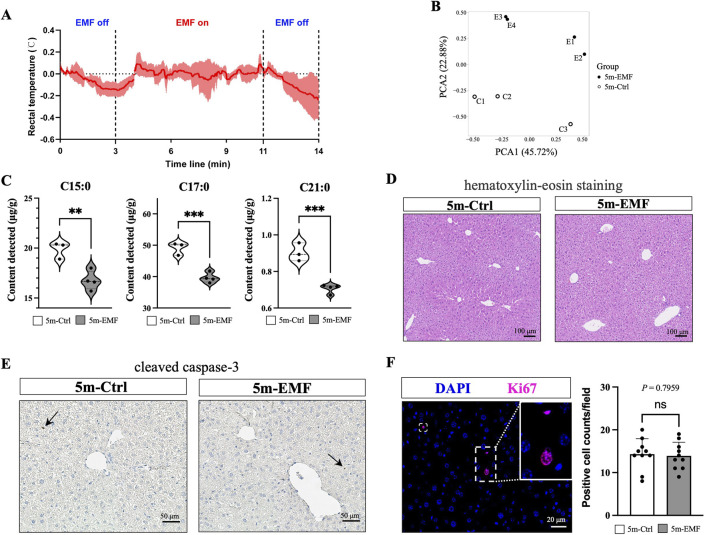
Functional and histological assessment of the murine liver after 5-month exposure to 2.45 GHz electromagnetic field (EMF). **(A)** Rectal temperature measurements of mice over a 14-min period, covering periods when EMF was off and on. **(B)** PCA analysis of liver tissue samples from 5m-Ctrl and 5m-EMF-exposed mice. **(C)** Contents of C15:0 (pentadecylic acid), C17:0 (margaric acid), and C21:0 (heneicosylic acid) in liver tissues (n = 4). **(D)** Representative images of H&E staining in liver tissues from 5m-Ctrl and 5m-EMF-exposed mice. Scale bars: 100 μm. **(E)** Immunohistochemical staining for cleaved caspase-3. Scale bars: 50 μm. **(F)** Immunofluorescence staining for Ki67 and quantification of Ki67-positive cells (*n* = 3, 10 fields per section). Scale bars: 20 μm. Values are presented as mean ± SD, and comparisons were made between 5m-Ctrl and 5m-EMF group. ns = not significant, n = replicates. Statistical significance is indicated as follows: *, *P* < 0.05, **, *P* < 0.01, ***, *P* < 0.001. All tests were two-tailed.

To prevent thermal effects, we established a long-term (5 months) but short-duration (6 min, daily) exposure strategy for the 2.45 GHz EMF radiation, which is commonly used in simulating wireless equipment (McKenzie et al., 2024). The measured body temperature suggests that the experimental conditions have a negligible effect on body temperature (Figure 1A). On day 150, plasma alkaline phosphatase (ALP) levels notably increased, and alanine aminotransferase (ALT), aspartate aminotransferase (AST), and cholinesterase (CHE) levels slightly increased, which suggested possible hepatocellular injury (Supplementary Figure S1). Considering that imbalances in hepatic lipid metabolism serve as sensitive biomarkers for liver damage, we performed a lipidomic analysis of the liver tissues collected from the control and exposed mice (Ctrl, *n* = 3; Exposure, *n* = 4). As shown in Figure 1B, PCA analysis demonstrated distinct separation between the 5m-EMF and 5m-Ctrl groups, demonstrating that the EMF exposure induced significant changes in the lipid profiles of the liver. The fatty acid (FA) profile showed significant reductions in the content of C_16_H_32_O_2_ (C15:0, pentadecylic acid), C_18_H_36_O_2_ (C17:0, margaric acid), and C_22_H_44_O_2_ (C21:0, heneicosylic acid), which are categorized as odd-chain saturated fatty acids (OCSFAs) (Figure 1C; Supplementary Figure S2).

Further, we conducted H&E staining to examine the potential histological alterations induced by EMF. No significant signs of necrosis, lipid droplet deposition, or inflammation were observed (Figure 1D). Additionally, tissue injury was investigated by staining for cleaved caspase-3, a widely used biomarker to detect early apoptosis, and only rare positive signals were detected (Figure 1E). Ki-67-expressing cells were also stained and counted to evaluate the overall proliferation status of the liver (Figure 1F). No differences in the counts of Ki67-positive cells were observed between the EMF exposure and control groups, suggesting that long-term EMF exposure exerted negligible effects on liver cell proliferation.

### 3.2 scRNA-seq identified multiple cell types in livers from control and EMF-Exposed mice

**FIGURE 2:**
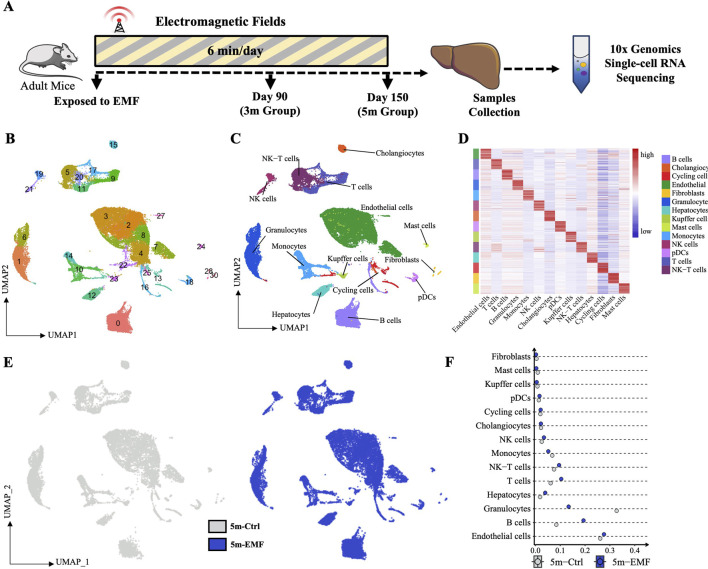
Single-cell transcriptomics of hepatic cells after EMF exposure. **(A)** Experimental design of the single-cell study. Mice were irradiated daily, and liver samples for scRNA-seq were collected on days 90 (3m group) and 150 (5m group). **(B)** UMAP visualization of cell distribution across the different groups, with cells colored by their recognized clusters. **(C)** UMAP plots of hepatic cells, visualizing 14 identified cell types. **(D)** Heatmap of the top 20 differentially expressed genes (DEGs) distinguishing individual cell types. **(E)** UMAP plots showing no significant cell type loss between the Ctrl and EMF groups. **(F)** Proportion of identified cell types of each condition in 5m group. pDCs: plasmacytoid dendritic cells.

To thoroughly elucidate the transcriptomic variations across diverse hepatic cell populations, and to explore the continuous or intermittent transcriptomic changes throughout the long-term exposure, we conducted scRNA-seq on the liver from the control (Ctrl, *n* = 3) and EMF-exposed mice at two time points (3m-EMF, *n* = 1; 5m-EMF, *n* = 2) (Figure 2A). We sequenced 63,466 cells at a mean read depth of 46 K reads per cell in accordance with the 10× Chromium protocol. In total, 55,553 cells were further analyzed after quality control and batch-effect removal. We identified and visualized 31 clusters using uniform manifold approximation and projection (UMAP) (Figure 2B). The following 14 heterogeneous cell types were further annotated according to the known lineage markers and identified cluster-specific genes (Figure 2C,D; Supplementary Figure S3A): endothelial cells (*Pecam1*, 27.88%), hepatocytes (*Alb*, 11.5%), cholangiocytes (*Epcam*, 1.88%), granulocytes (*Csf3r*, 15.77%), Kupffer cells (*C1qc*, 0.65%), monocytes (*Chil3*, 5.09%), B cells (*Cd79a*, 16.64%), T cells (*Cd3d*, 7.32%), NK cells (*Ncr1*, 2.45%), NK-T cells (*Nkg7*, 6.96%), fibroblasts (*Pdgfrb*, 0.32%), mast cells (*Osm*, 0.49%), dendritic cells (*Siglech*, 1.34%), and cycling cells (*Top2a*, 1.72%). The split UMAPs showed no obvious emergence or depletion of cell populations after 3 or 5 months of EMF exposure (Figure 2E; Supplementary Figure S3B), whereas a detailed analysis of cell proportion indicated an increase in the proportions of B cells, hepatocytes, and T cells after up to 5 months of exposure and a decrease in the proportion of granulocytes (Figure 2F; Supplementary Figure S3C).

**FIGURE 3:**
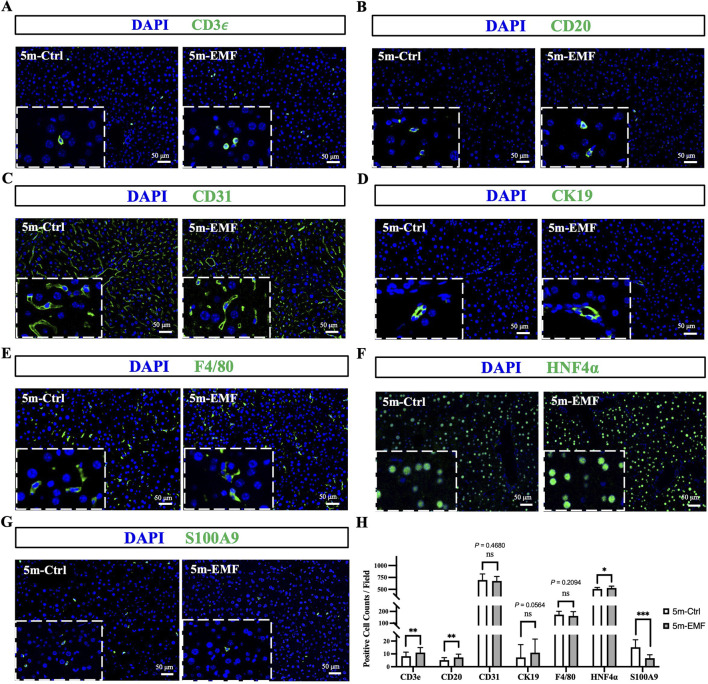
Immunofluorescence staining of key liver cell markers in 5m-Ctrl and 5m-EMF groups. **(A–G)** Representative immunofluorescence images for CD3e (T cells), CD20 (B cells), CD31 (endothelial cells), CK19 (cholangiocytes), F4/80 (Kupffer cells), HNF4α (hepatocytes), and S100A9 (granulocytes). **(H)** Quantification of positive cells per field. Scale bars: 50 μm. The data that follows a normal distribution were analyzed using an independent t-test, while non-normally distributed data were analyzed using the Mann-Whitney U test. Values are presented as mean ± SE, and comparisons were made between 5m-Ctrl and 5m-EMF group. ns = not significant. Statistical significance is indicated as follows: *, *P* < 0.05, ***, *P* < 0.001. All tests were two-tailed.

Furthermore, we validated the above results with *in situ* tissue staining. Cell type-specific marker staining revealed that the percentages of endothelial cells (CD31^+^) and cholangiocytes (CK19^+^) remained consistent between the exposure and control groups, whereas 5-month EMF exposure modestly increased the proportions of T cells (CD3e^+^), B cells (CD20^+^), and hepatocytes (HNF4a^+^) and reduced the proportion of granulocytes (S100A9^+^) (Figures 3A–H, and Supplementary Figures S4A–S4H).

### 3.3 Evaluating EMF sensitivity of multiple hepatic cells at single-cell resolution

**FIGURE 4:**
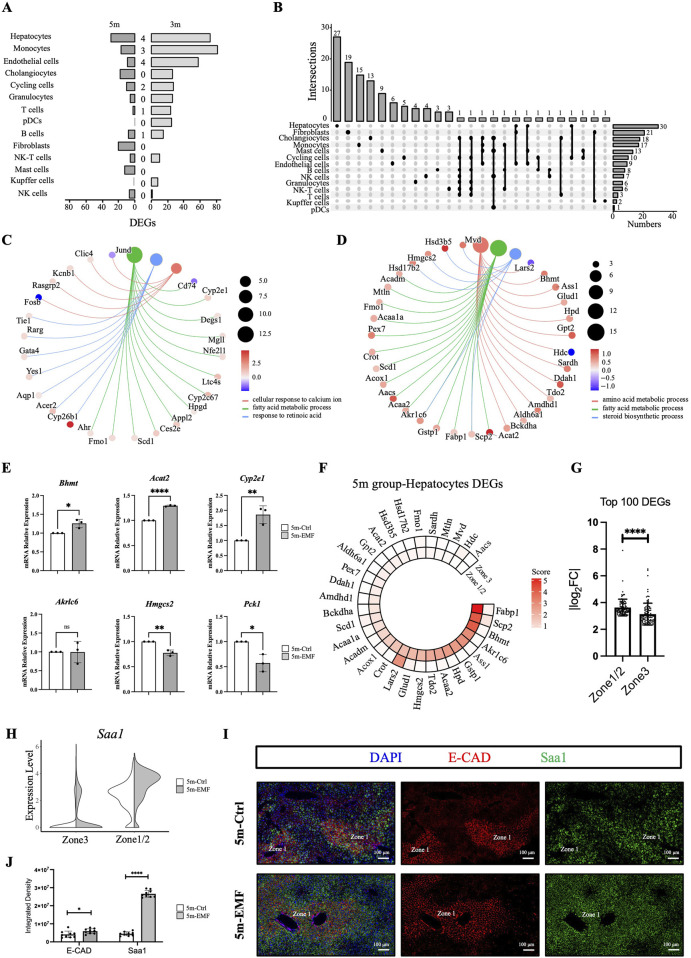
EMF-induced transcriptome alterations across hepatic cell populations and zonation. **(A)** Numbers of significant DEGs (|log_2_FC| > 0.5 and adj. *p* < 0.05) of 14 populations and intersections between the 3m and 5m groups. **(B)** UpSet plot showing the significant DEGs of 14 populations in the 5m group, with the number of cell type-unique DEGs labeled at the top. **(C,D)** Cnetplots illustrating the affected biological processes and related DEGs identified by GO analysis in hepatocytes from the 3m group **(C)** and the 5m group **(D)**. Color map represents log2FC (log2 fold change). **(E)** Expression of *Bhmt, Acat2, Cyp2e1, Akr1c6, Hmgcs2,* and *Pck1* in liver tissues measured by qPCR. **(F)** Circle heatmaps showing the average expression of the pathway-enriched differentially expressed genes (DEGs) in hepatocytes from the 5m group. **(G)** Zonation distribution analysis of the top 100 DEGs (ranked by absolute log2FC) in the 5m groups. Each dot in the bar plot represents an individual DEG. **(H)** Violin plot of *Saa1* expression in zonation-defined hepatocytes in the 5m groups. **(I)** Representative images of *Saa1* (green) and E-cadherin (E-CAD, red) staining in liver sections from the 5m group. *Saa1* expression was detected by RNA *in situ* hybridization, E-CAD was stained by antibody. Scale bars: 100 μm. **(J)** Quantitative analysis of the fluorescence density for *Saa1* and E-cadherin between 5m-Ctrl and 5m-EMF group (*n* = 3 mice per group). Values are presented as mean ± SD. Statistical significance is indicated as follows: ^*^, *P* < 0.05, ^**^, *P* < 0.01, ^****^ *P* < 0.0001, ns = not significant.

To further investigate whether different types of hepatic cells show various sensitivities to long-term EMF exposure, we focused on the EMF-induced transcriptomic changes in the cell populations identified above. Assessment based on the number of DEGs at 3 months showed that monocytes, hepatocytes, and endothelial cells exhibited high sensitivity and significant responses to EMF radiation (Figure 4A). Further analysis of the response patterns of the different cell types revealed that 12 of the 14 cell types had three or fewer common DEGs between the two tested time points (Figure 4A), suggesting that the gene expression alterations in hepatic cells were discontinuous during the exposure period. To evaluate whether the alterations are progressive or adaptive, we performed pseudotime analysis on the hepatocytes across different groups and time points. We observed that the overall transcriptional characteristics of hepatocyte clusters remained relatively stable, with no significant movement of clusters along the trajectory axis (Supplementary Figure S5A). We concluded that the transcriptional changes in hepatocytes under these experimental conditions are presumably adaptive responses. Moreover, no more than three common DEGs were found among the different cell types on day 150 (Figure 4B), indicating that each cell type had an exclusive response to EMF radiation.

Considering that hepatocytes play critical roles in executing essential liver functions, we explored their potential functional alterations by performing Gene Ontology (GO) analysis of the hepatocyte-derived DEGs. On day 90, FA metabolism was significantly enriched (Figure 4C). This metabolic alteration persisted through day 150, during which time the amino acid metabolism and steroid biosynthesis were also disturbed (Figure 4D). The transcriptional of the involved genes, including *Bhmt*, *Acat2*, *Cyp2e1*, *Akr1c6*, *Hmgcs2*, and *Pck1*, were further tested through qPCR quantification (Figure 4E). These genetic alterations might explain the underlying mechanisms responsible for the EMF-induced FA profiling disturbance in the liver.

### 3.4 Zonation-defined metabolic function alternation of hepatocytes following long-term EMF exposure

Multiple hepatic cells exhibit spatial heterogeneity along the portal–central axis in the liver lobules. However, the zonation patterns of lipid and steroid metabolism remain controversial (Schleicher et al., 2015). To precisely explore the zonal sensitivity of hepatic cells to long-term EMF exposure, we further annotated hepatocyte clusters with the acknowledged zonation-associated maker genes (Halpern et al., 2017) (Supplementary Figure S5B). Cluster 12 was designated as zone 1/zone 2 (periportal or mid-lobule) hepatocytes, involved in amino acid catabolism (*Gls2*, *Hal*, *Sds*) and ion homeostasis regulation (*Hamp*); clusters 4 and 22 were designated as zone 3 (pericentral) hepatocytes, characterized by detoxification functions (*Glul*, *Gstm2*) (Supplementary Figures S5C,D). Then we probed the zonal expression patterns of the pathway-enriched DEGs in hepatocytes. The DEGs on day 90 were distributed across the entire lobule, with no tendency toward higher expression in any zonation (Supplementary Figure S5E). Nevertheless, the DEGs on day 150 showed notable expression biases in zone 1/zone 2 hepatocytes (Figure 4F). Quantitative analysis of the top 100 DEGs by absolute log2 fold change further demonstrated that zone 1/zone 2 hepatocyte exhibited significantly greater changes after EMF exposure (Figure 4G). These results indicated high sensitivity of the periportal and mid-lobule hepatocytes to long-term EMF exposure. To further validate the region-defined sensitivity, we selected the most significantly upregulated gene (*SAA1*, Supplementary Figure S5F) in the 5m-EMF mice, along with the zone 1 hepatocyte marker E-cadherin (E-CAD) to perform *in situ* co-staining. In consistent with the data analysis result (Figure 4H), the staining results showed that *SAA1* was essentially co-expressed with E-CAD in zone 1 in the control mice but could be strongly induced in zone 1/zone 2 hepatocytes in the radiated mice (Figures 4I,J). These results indicated that hepatocytes exhibited a region-defined sensitivity to long-term EMF exposure.

### 3.5 Impact and zonal specificity of EMF on liver endothelial cells

**FIGURE 5:**
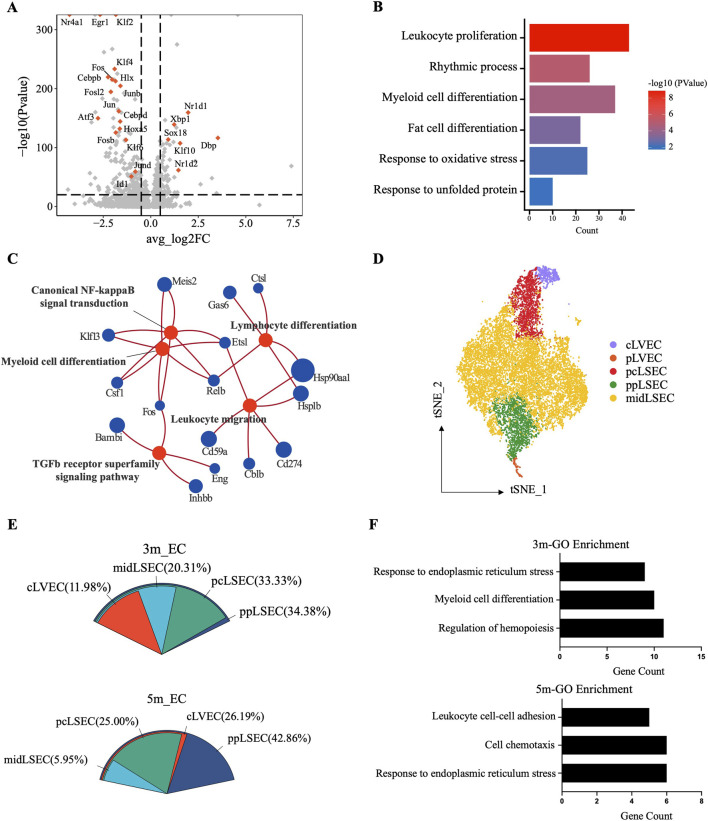
Single-cell transcriptomic analysis of the endothelial cell responses to EMF. **(A)** Volcano plot showing significant DEGs of endothelial cells between exposed and control mice in the 3m group. Colored dots denote transcription factor (TF)-coding genes. **(B)** Barplot illustrating the affected biological processes in liver endothelial cells after 3-month exposure. **(C)** Enrichment analysis of the DEGs with the GO pathways database in the 5m group. **(D)** t-SNE plot of the zonation-annotated endothelial cells, with colored dots denoting cells from each zonation. **(E)** Proportional distribution of DEGs in region-annotated endothelial subclusters from the 3m and 5m groups. **(F)** Enriched GO terms (p < 0.05) of DEGs derived from ppLSEC subclusters.

As one of the EMF-sensitive cell types, endothelial cells were analyzed for their response to long-term EMF exposure. We found that most of the top-ranked DEGs from the 3m group were transcription factors (TFs, Figure 5A), with functions involved in response to unfolded protein (*Atf3*) and oxidative stress (*Fos*), leukocyte proliferation (*Junb*), myeloid cell (*Fos*, *Junb*), and fat cell (*Cebpd*) differentiation, and rhythmic process (*Dbp*) (Figure 5B). GO analysis of the DEGs in the 5m group also revealed that leukocyte differentiation and migration were influenced. Moreover, canonical NF-kappaB and TGFb receptor superfamily signaling pathways might serve as the potential mechanism (Figure 5C). Therefore, we speculated that the long-term EMF affected the interactions between immune cells and endothelial cells.

Considering that liver endothelial cells can also be well annotated based on their zonal-specific markers (Halpern et al., 2018; Su et al., 2021; Strauss et al., 2017), we explored the zonal sensitivity of endothelial cells to EMF. The identified endothelial cells were annotated as mid-zonal liver sinusoidal endothelial cells (midLSEC), pericentral liver sinusoidal endothelial cells (pcLSEC), periportal liver sinusoidal endothelial cells (ppLSEC), central liver vessel endothelial cells (cLVEC), and portal liver vessel endothelial cells (pLVEC) according to the recognized landmarks (Figure 5D; Supplementary Figure S6A). The subcluster proportion between the control and exposed groups basically remained constant (Supplementary Figure S6B). Subsequently, we determined DEGs in zone-specific ECs and observed that the highest number of DEGs was associated with ppLSEC (Figure 5E). Correspondingly, the GO analysis of the ppLSEC-derived DEGs also indicated endoplasmic reticulum stress and altered immune regulatory functions, which were consistent with the changes in the entire population of endothelial cells (Figure 5F). All the above suggested a greater sensitivity of ppLSEC to long-term RF-EMF exposure.

### 3.6 Functional assessment of the immune cells in EMF-Radiated liver

**FIGURE 6:**
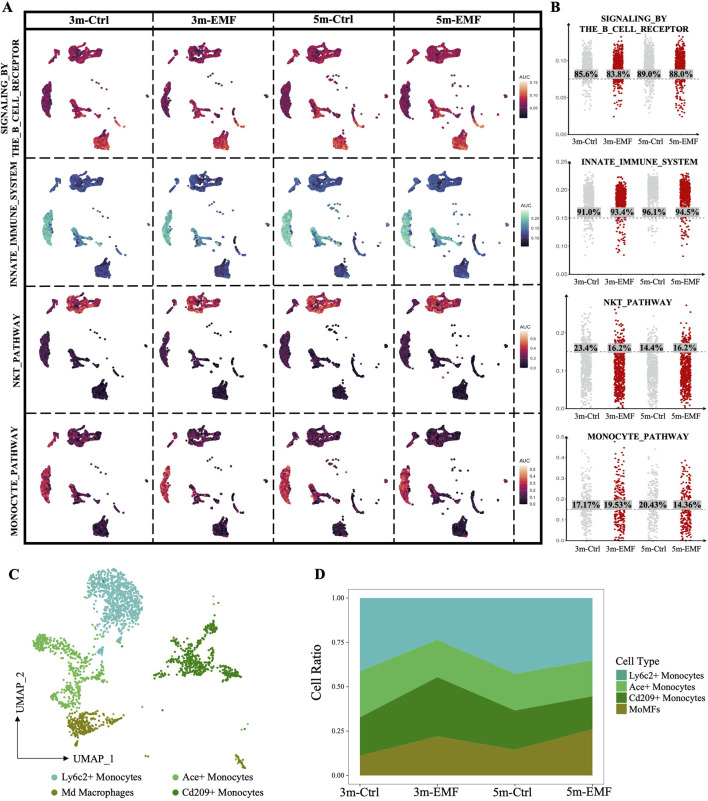
Assessment of the EMF-induced functional alterations in immune cells. **(A)** AUCell scores of the curated pathways were visualized on UMAPs, highlighting functional alterations in immune cells in response to EMF exposure. **(B)** Columnar scatter plots showing the distribution of AUCell scores across the 3m-Ctrl, 3m-EMF, 5m-Ctrl, and 5m-EMF groups. The percentage of cells with scores higher than the median is labeled. **(C)** UMAP visualization of the four identified monocyte subclusters, with different subclusters colored distinctively. **(D)** Proportional distribution of monocyte subclusters in each group.

As shown in Figure 4A, the immune cells in the liver also showed high sensitivity to EMF. The DEGs derived from the B cells, granulocytes, monocytes and NK-T cells in the 5m group, are displayed in Supplementary Figure S7A. The GO analysis of the DEGs demonstrated that biological processes related to leukocyte migration and proliferation were commonly affected in immune cells (Supplementary Figure S7B), which was consistent with the alterations in immune regulatory function in endothelial cells. Furthermore, we calculated the AUCell scores of the function-related pathways to evaluate the alterations in immune cells. By calculating the proportion of immune cells with the respective activated canonical pathways (B cells: signaling by the B cell receptor; granulocytes: innate immune system; NK-T cells: NKT pathway; monocytes: monocyte pathway), we found that long-term EMF exposure barely affected the basic functions of B cells and granulocytes but exerted detrimental effects on NK-T cells and monocytes, especially on monocytes, as evidenced by the decreased proportion of activated monocytes in the 5m group (Figures 6A,B). Furthermore, we identified four subpopulations within the mononuclear phagocyte system (MPS) and annotated the cell clusters as Ly6c2^+^ Monocytes, Ace^+^ monocytes, Cd209a^+^ monocytes, and Adgre1^+^ monocyte-derived macrophages (MoMFs) (Figure 6C; Supplementary Figure S7C). After RF-EMF exposure, the proportion of MoMFs was elevated while that of Ly6c2^+^ classic monocytes was declined (Figure 6D), implying that the differentiation of monocytes might be affected by EMF. To further explore the functional alteration triggered by the shift from Ly6c2^+^ monocytes to MoMFs, we performed cytokine expression analysis on the MPS populations. As shown in the heatmap in Supplementary Figure S7D, significantly, the expression of anti-inflammatory cytokines Il10 and Tgfb1 increased in 5m-EMF group. Therefore, we further performed CellChat analysis focusing on the role of Tgfb1. This cell-cell communication analysis demonstrated echanced crrosstalks between B/T cells and Ly6C2^+^ monocytes via TGFβ signaling in the 5m-EMF group, suggesting the shift from Ly6c2^+^ monocytes to MoMFs contributed to the altered intercellular communication (Supplementary Figure S7E).

## 4 Discussion

Although the cellular and molecular mechanisms underlying the effects of EMF exposure remain unclear, previous studies have established that EMFs exert deleterious effects on the nervous, productive, and hematological systems (Liu et al., 2024; Lai, 2021). The liver is considered non-sensitive to ionizing and non-ionizing radiation because hepatocytes are characterized by low turnover and high regenerative capacity (Gilgenkrantz and Collin de l’Hortet, 2018; Guo et al., 2022; He et al., 2021). In the present study, we explored EMF-induced liver alterations at the transcriptomic level and at single-cell resolution by using scRNA-seq. Finally, we precisely described the spatiotemporal effects of long-term electromagnetic radiation on various hepatic cells (Supplementary Figure S8).

Hepatocytes are the major functional cells of the liver and play essential roles in the metabolism of glucose, lipids, and proteins. Previous studies have shown that EMF increases the expression of genes related to glucose transport and the tricarboxylic acid cycle in yeast (Lin et al., 2016). Here, we also observed the increased blood glucose and lactate levels of the mice after long-term EMF exposure (unpublished data). More importantly, we revealed an obvious reduction in the contents of OCSFAs, such as C15:0 and C17:0, in the liver after EMF exposure. As OCSFAs repair mitochondrial function and reduce proinflammatory or profibrotic states (Venn-Wats et al., 2020), continuous monitoring of the circulating concentration of OCSFAs should be considered in coping with long-term exposure to EMFs.

Moreover, by annotating hepatocytes with regionally expressed genes, we observed differences in hepatocyte sensitivity across the liver lobules. In addition to hepatocytes, cholangiocytes, another type of epithelial cells, also showed significant transcriptional changes. Cholangiocytes are in the portal vein region of the liver, which supports the higher sensitivity of zone 1 in the liver tissue. Zonal disparities in hepatocyte sensitivity are likely influenced by distinct microenvironments, especially the high oxygen tension and nutrient abundance in zone 1/2 (Cunningham and Porat-Shliom, 2021), which promotes heightened metabolic activity and oxidative phosphorylation. These metabolically active hepatocytes may be particularly susceptible to EMF-induced mitochondrial dysfunction (Rana et al., 2024), potentially resulting in increased reactive oxygen species (ROS) production and cellular stress.

In the liver, endothelial cells are functionally and spatially heterogeneous, and participate in circulating antigen removal, vascular tone regulation, and immune cell functions. Zonation-specific changes in endothelial cells have been observed in multiple liver injuries (Koch et al., 2021). In the present study, we demonstrated a relatively higher vulnerability of peri-portal endothelial cells. Zone 1 endothelial cells regulate immune cell adhesion and migration (Strauss et al., 2017), together with dysregulated migration pathways in immune cells, which might explain the observed infiltration of T and B cells in the liver tissues. Moreover, we identified significant downregulation of TFs, including Klf2, Klf4, Fos, Fosb, Jun, and Junb, in endothelial cells. These TFs are deeply involved in the regulation of nitric oxide production and are also downregulated in cirrhotic livers (Su et al., 2021), suggesting that the vascular tone alteration after EMF exposure warrants close attention. However, whether long-term EMF exposure induces liver fibrosis or increases vulnerability to stimuli requires further investigation.

The immune microenvironment of the liver consists of diverse populations of myeloid cells and lymphocytes that play various roles in the response to endogenous and exogenous injuries (Markose et al., 2018). In the present study, monocytes were relatively sensitive to EMF, with their intrinsic functions and differentiation disturbed under long-term EMF exposure. Monocytes are the largest leukocytes and are rich in lysosomal granules (Boyette et al., 2017); their higher sensitivity is consistent with the report of Shi et al. that the cellular effects of electromagnetic waves are the sum of the constituents of cells and are related to the cell radius (Lu et al., 2023). However, the specific mechanisms warrant further investigation.

In summary, using single-cell transcriptome sequencing, we conducted an in-depth analysis of the temporal-spatial changes in the gene expression of various hepatic cells induced by long-term EMF exposure and systematically revealed the biological effects of EMF on the liver. Nevertheless, this study has limitations, including the lack of clinical validation of the observed liver functional changes, the limited single-cell transcriptomic sample size, and the simplex genetic background of the mouse models. We believe that our research lays a foundation for broadening our understanding of the biological effects of EMF and enhances the awareness of surveillance and protection against its potential health risks.

## 5 Opening up

This study performed a single-cell resolved investigation into the spatiotemporal effects of long-term 2.45 GHz EMF exposure on liver. The results expanded our understanding of the biological effects of electromagnetic radiation and provided novel insights into the response patterns of the liver to the public and environmental health risk factors.

## Data Availability

The datasets presented in this study can be found in online repositories. The names of the repository/repositories and accession number(s) can be found in the article/Supplementary Material.

## Abbreviations

EMF, Electromagnetic Field; DEGs, Differentially Expressed Genes; GO, Gene Ontology; OCSFAs, Odd-Chain Saturated Fatty Acids; qPCR, Quantitative Polymerase Chain Reaction; SAA1, Serum Amyloid A1; LSEC, Liver Sinusoidal Endothelial Cells; ppLSEC, Periportal Liver Sinusoidal Endothelial Cells; midLSEC, Mid-Zonal Liver Sinusoidal Endothelial Cells; pcLSEC, Pericentral Liver Sinusoidal Endothelial Cells; cLVEC, Central Liver Vessel Endothelial Cells; pLVEC, Portal Liver Vessel Endothelial Cells; Atf3, Activating Transcription Factor 3; Fos, Proto-oncogene Fos; Junb, Jun B Proto-oncogene; Cebpd, CCAAT/Enhancer Binding Protein Delta; Dbp, D-box Binding Protein; NF-kappaB, Nuclear Factor Kappa B; TGFb, Transforming Growth Factor Beta; Ly6c2, Lymphocyte Antigen 6 Complex, Locus C2; Ace, Angiotensin-Converting Enzyme; Cd209a, C-Type Lectin Domain Family 4 Member A; Adgre1, Adhesion G Protein-Coupled Receptor E1 (F4/80); MoMFs, Monocyte-Derived Macrophages.
