# Supplementary material for: Single-cell analysis reveals the spatiotemporal effects of long-term electromagnetic field exposure on the liver
Source: Front Cell Dev Biol. 2025 Jun 27;13:1579121. doi: 10.3389/fcell.2025.1579121 (PMC12245793; doi:10.3389/fcell.2025.1579121)
Supplement: Supplementary file 1 [file DataSheet1.pdf]

Supporting Information for

Single-Cell Analysis Reveals the Spatiotemporal Effects of Long-Term Electromagnetic Field Exposure on the Liver

Mingming Zhang [a], Zhichun Lv [a], Lingping Zhao [a], Quan Zeng [a], Yunqiang Wu [a], Junnian Zhou [a], Jiafei Xi [a], Xuetao Pei [a], Haiyang Wang [a, *], Changyan Li [a, *], Wen Yue [a, *]

[a] Beijing Institute of Radiation Medicine, Beijing 100850, China

*Corresponding authors: Haiyang Wang, Changyan Li, Wen Yue.

E-mail: beckyhy0403@126.com (H.W.); fmmli@163.com (Changyan Li); yuewen@bmi.ac.cn (Wen Yue)

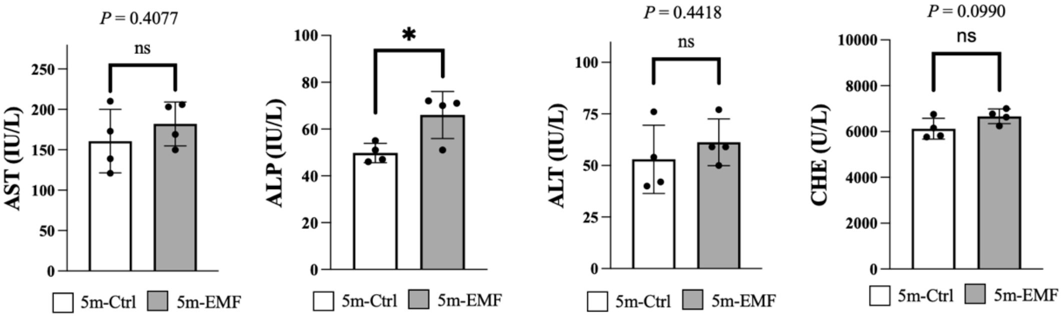

Figure S1. Assessment of Serum Markers of Hepatocyte Injury in Mice after EMF Exposure.

Concentrations of ALP, ALT, AST, and CHE in mouse serum (n = 4). Statistical analysis was performed using an independent t-test for normally distributed data and the Mann-Whitney U test for non-normally distributed data. Power values of serum indicators are as follows: 0.1144 (AST), 0.1051(ALT), 0.6175 (ALP), and 0.3624 (CHE), with Alpha value set at 0.05.

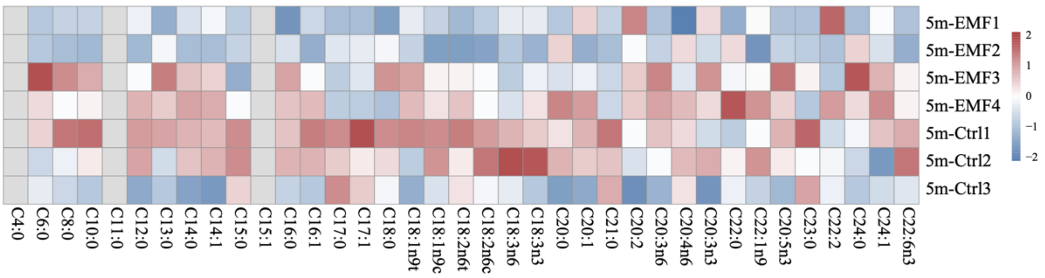

Figure S2. Assessment of Liver Fatty Acid Content after EMF Exposure.

Heatmap showing the content of detected fatty acids across the 5m-Ctrl and 5m-EMF groups.

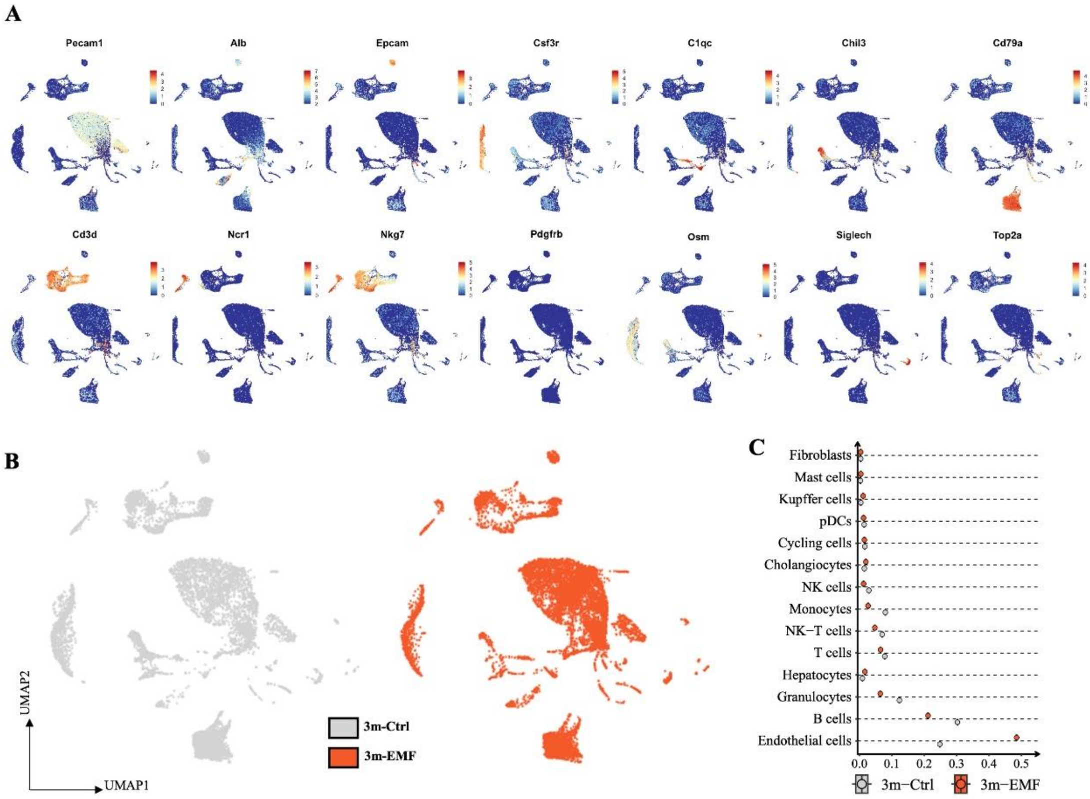

Figure S3. Cell marker expression and cell proportion in liver samples from 3m-EMF exposed mice. (**A**) UMAP plots visualizing the expression of specific genes in the map, highlighting cell type-specific gene expression. (**B**) UMAP visualization showing the distribution of cells in the 3m-Ctrl and 3m-EMF group. (**C**) Proportion of identified cell types in each condition of 3m group.

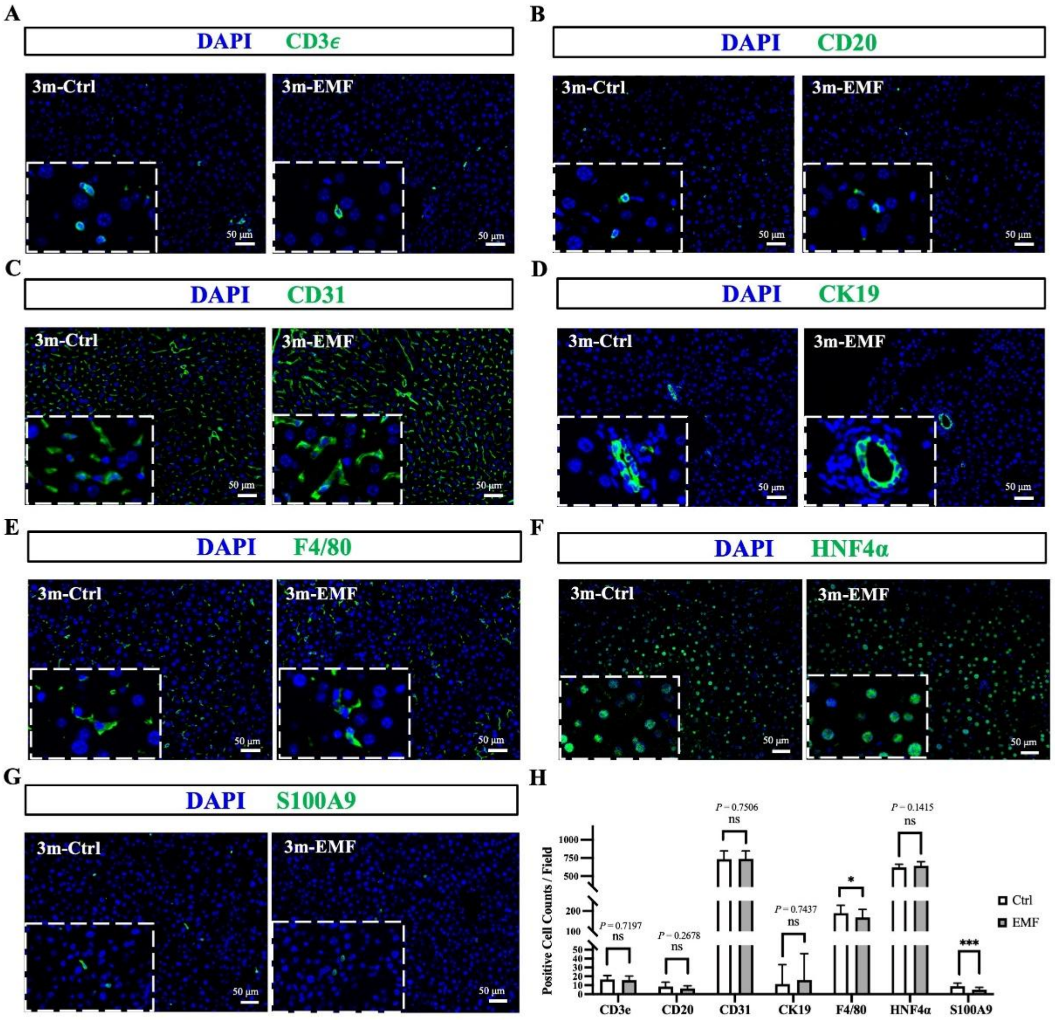

Figure S4. Immunofluorescence staining of liver cell markers after 3-month EMF exposure. Immunofluorescence staining of key liver cell markers in 3m-Ctrl and 3m-EMF groups. (**A-G**) Representative immunofluorescence images for CD3e (T cells), CD20 (B cells), CD31 (endothelial cells), CK19 (cholangiocytes), F4/80 (Kupffer cells), HNF4α (hepatocytes), and S100A9 (granulocytes). (**H**) Quantification of positive cells per field presented below each marker. Scale bars: 50 μm. The data that follows a normal distribution were analyzed using an independent t-test, while non-normally distributed data were analyzed using the Mann-Whitney U test. Values are presented as mean ± SE, and comparisons were made between 3m-Ctrl and 3m-EMF group. ns = not significant. Statistical significance is indicated as follows: *, $P < 0.05$, ***, $P < 0.001$. All tests were two-tailed.

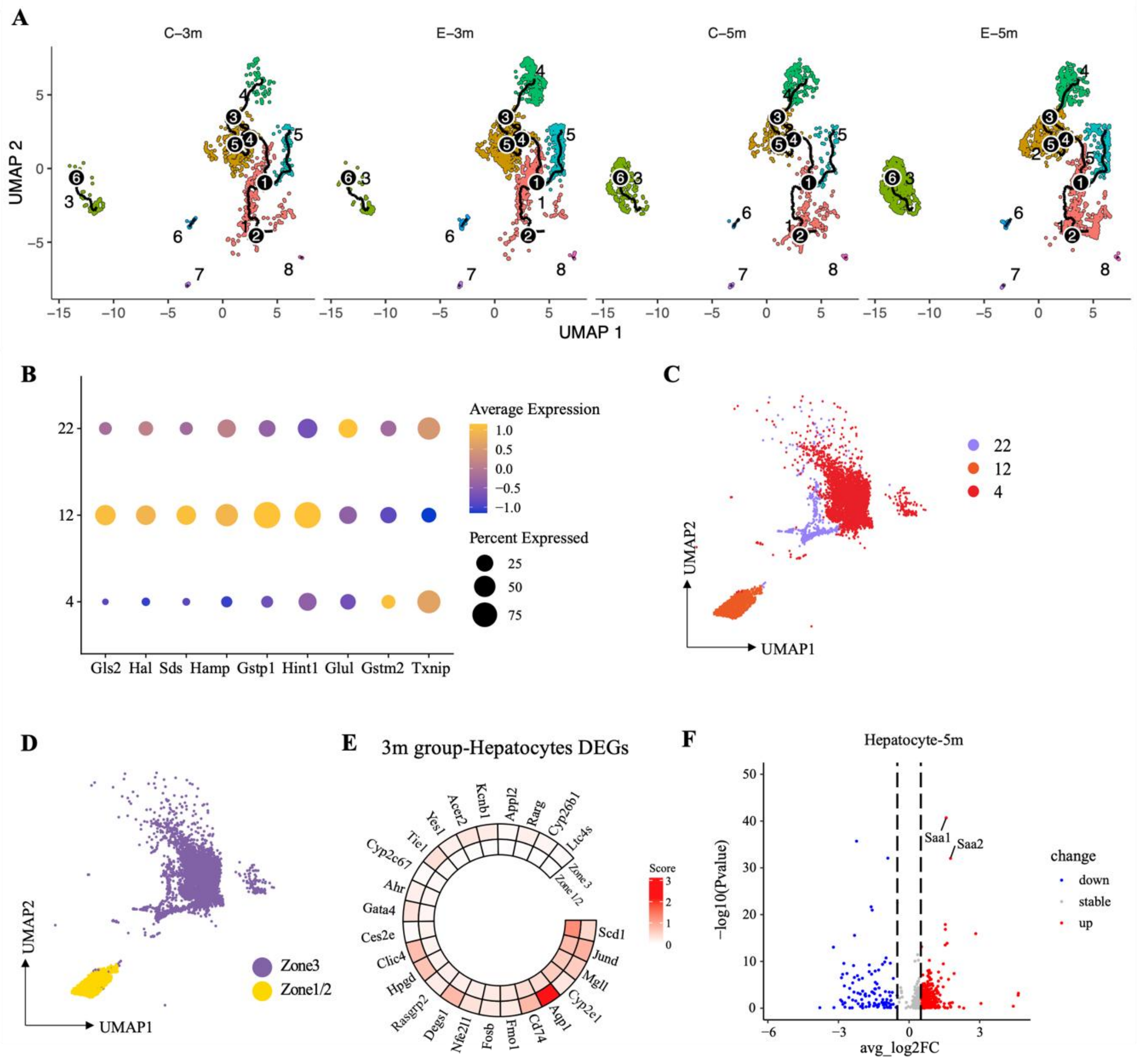

Figure S5. EMF induced zonation-defined alterations in hepatocytes. (**A**) Trajectory analysis of hepatocytes based on original clusters, performed by Monocle3. Colors represent different clusters. Numbers represent branching nodes. The distance along the trajectory indicates the transcriptional similarity between different cells. (**B**) Bubble plot showing the average expression level of the representative hepatocyte zonation markers in hepatocyte subclusters. (**C-D**) UMAP visualization of hepatocytes across the Ctrl and EMF groups, color-coded by their subclusters (**C**) and designated zonations (**D**) respectively. (**E**) Circle heatmaps showing the average expression of the pathway-enriched differentially expressed genes (DEGs) in hepatocytes from the 3m group. (**F**) Volcano plot showing significant differentially

expressed genes (DEGs) in hepatocytes between 5m-Ctrl and 5m-EMF groups, with the threshold for significance indicated.

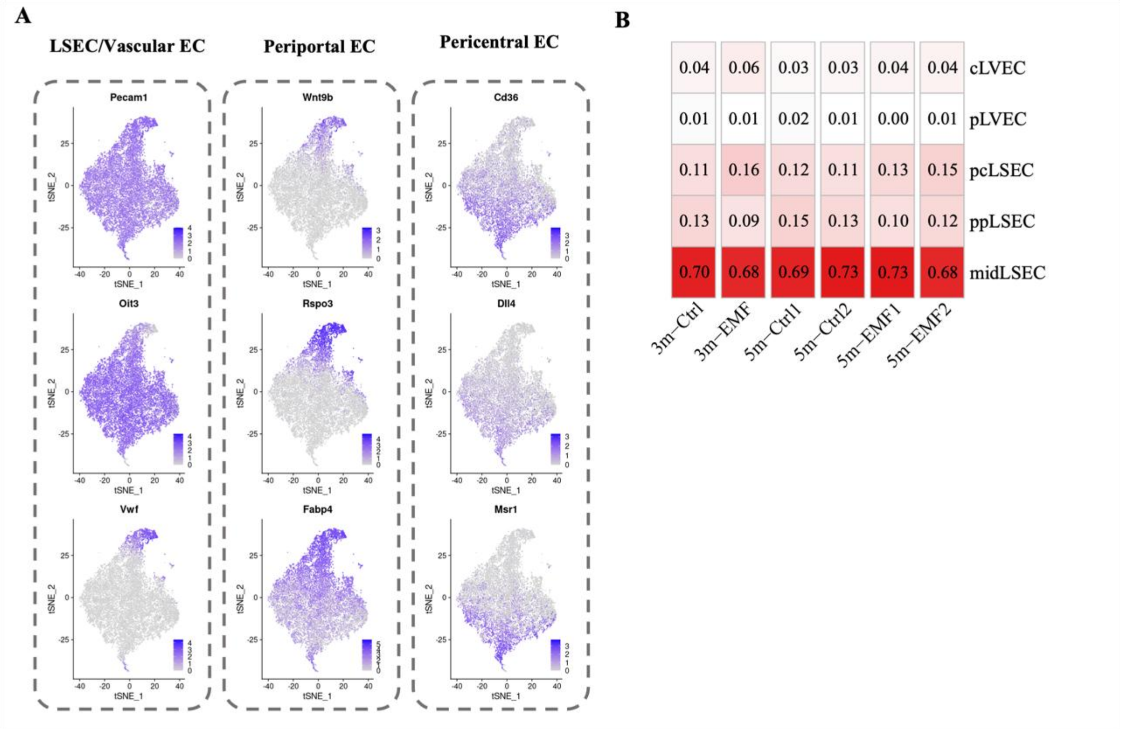

Figure S6. Annotations of endothelial cells and proportion analysis. (**A**) UMAP plots visualizing the expression of specific endothelial cell markers in different endothelial cell subclusters (LSEC/Vascular EC, Periportal EC, and Pericentral EC). (**B**) Proportional distribution of cell numbers in region-annotated endothelial subclusters from the 3m and 5m groups.

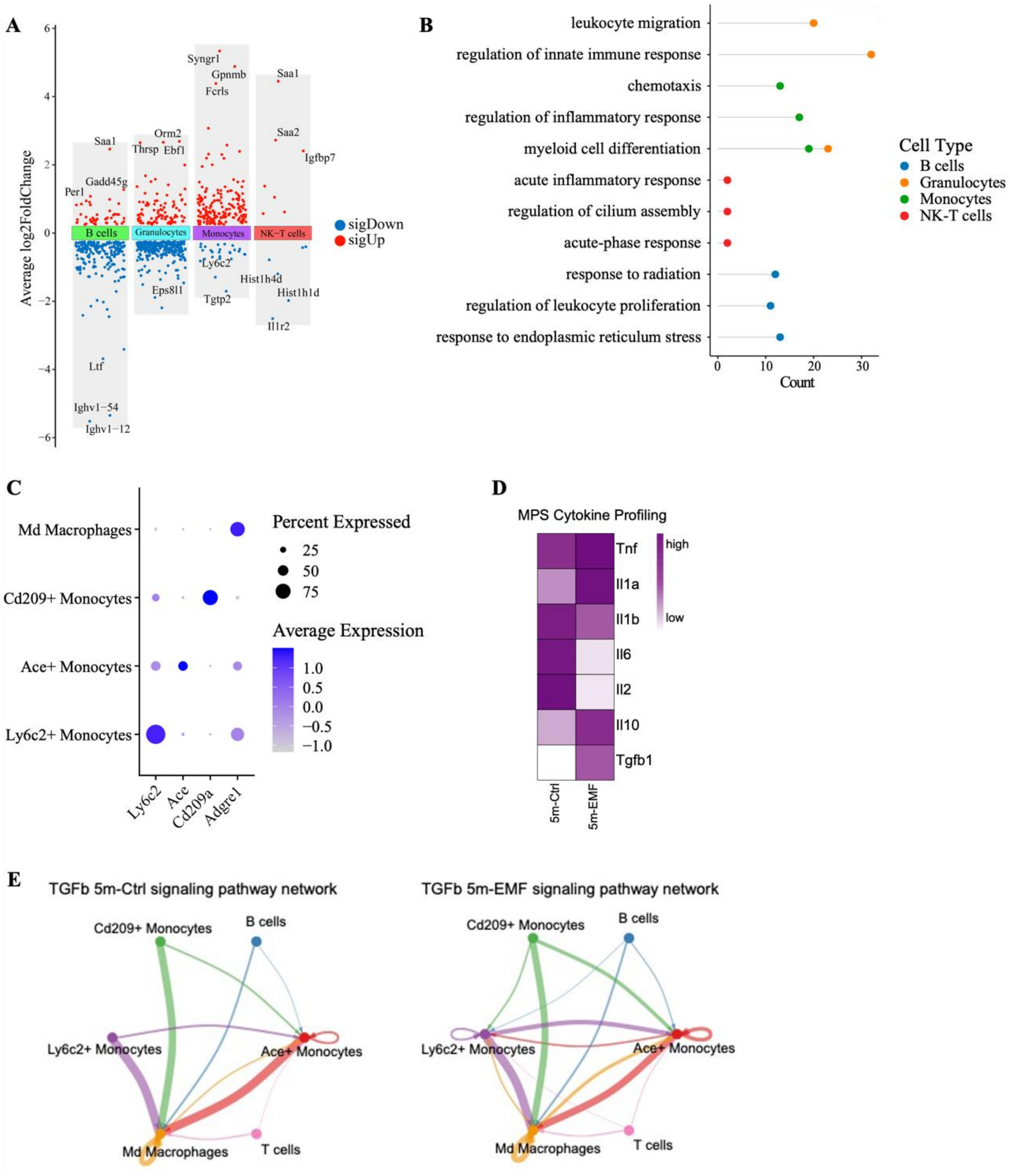

Figure S7. Differential Gene Expression and Functional Enrichment Analysis Among Immune Cells in the 5m Group. (**A**) DEGs in B cells, granulocytes, monocytes, and NK-T cells in the 5m group. The top five upregulated and downregulated genes determined by |average log2FC| in each cell type were labeled. (**B**) GO enrichment analysis of the DEGs in B cells, granulocytes, monocytes, and NK-T cells in the 5m group. (**C**) Bubble plot showing the average expression of the representative subtype markers of monocytes subclusters. (**D**) Heatmap illustrates the expression levels of key cytokines within the MPS across different groups. Color

intensity represents the relative expression level of each cytokine. (**E**) Network plots visualizing the inferred TGFβ signaling pathway interactions among different immune cell subtypes (Ly6c2^{+} Monocytes, Ace^{+} Monocytes, Cd209^{+} Monocytes, Md Macrophages, B cells, T cells) in the Control and EMF-exposed groups in 5m. The nodes represent cell subtypes, and the edges represent inferred signaling strength.

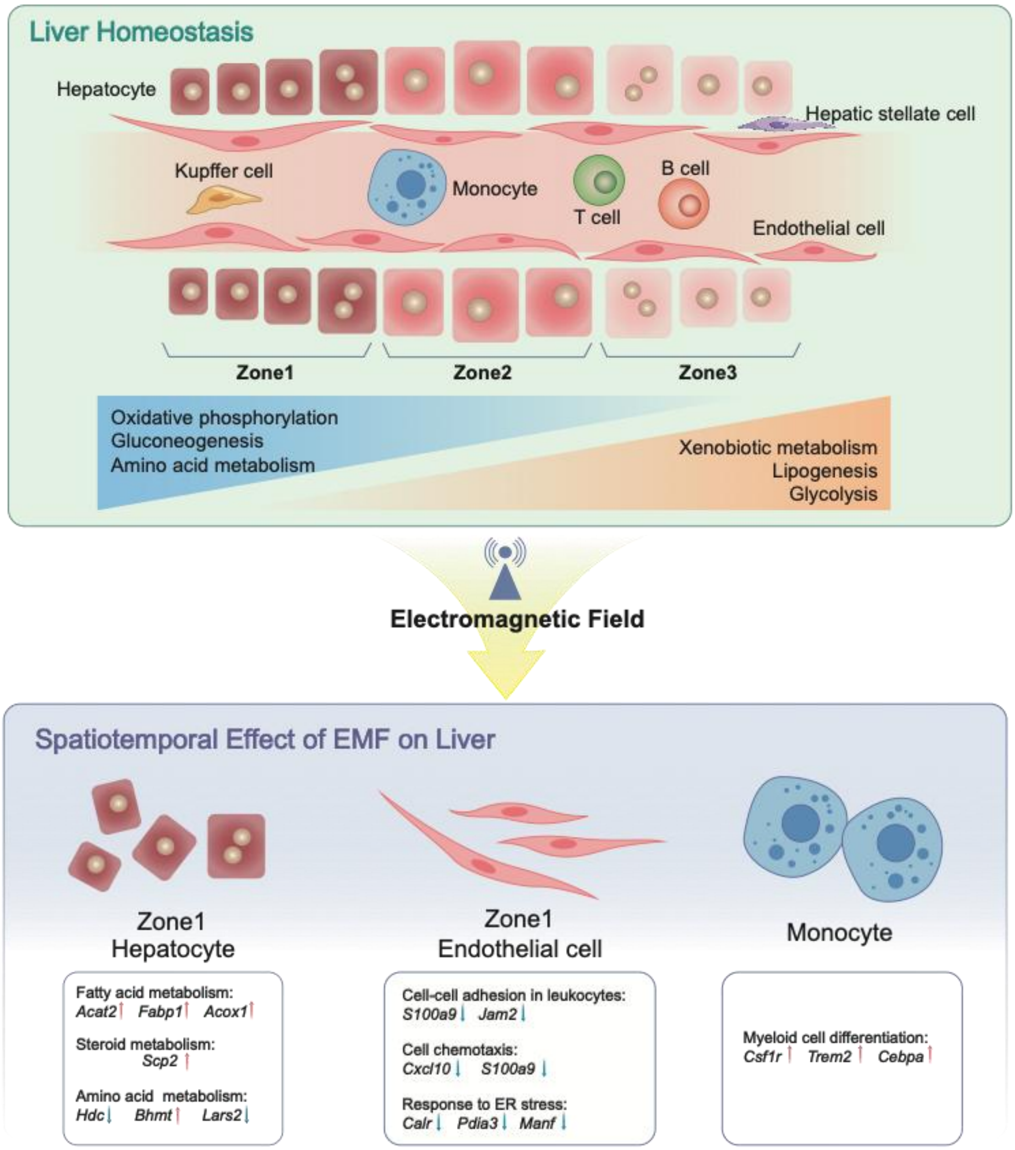

Figure S8. Schematic Illustration for the Region-defined Effects of Long-term Electromagnetic Radiation on Various Hepatic Cells.
